# A Review of Newly Diagnosed Glioblastoma

**DOI:** 10.3389/fonc.2020.574012

**Published:** 2021-02-05

**Authors:** Bryan Oronsky, Tony R. Reid, Arnold Oronsky, Navjot Sandhu, Susan J. Knox

**Affiliations:** ^1^ Department of Clinical Research, EpicentRx, San Diego, CA, United States; ^2^ Department of Medical Oncology, UC San Diego School of Medicine, San Diego, CA, United States; ^3^ Department of Clinical Research, InterWest Partners, Menlo Park, CA, United States; ^4^ Department of Radiation Oncology, Stanford University School of Medicine, Stanford, CA, United States

**Keywords:** ****glioblastoma, radiation therapy, high-grade gliomas, cancer, brain tumors

## Abstract

Glioblastoma is an aggressive and inevitably recurrent primary intra-axial brain tumor with a dismal prognosis. The current mainstay of treatment involves maximally safe surgical resection followed by radiotherapy over a 6-week period with concomitant temozolomide chemotherapy followed by temozolomide maintenance. This review provides a summary of the epidemiological, clinical, histologic and genetic characteristics of newly diagnosed disease as well as the current standard of care and potential future therapeutic prospects.

## Introduction

Glioblastoma, a World Health Organization grade IV astrocytoma, with an incidence in North America of 5.0 per 100,000 population, representing 15 to 20% of all primary intracranial neoplasms, in adults (1), is highly aggressive, with an unusually dismal prognosis (death typically results in the first 15-16 months after diagnosis) (2), with a 5-year survival rate of 5%. These tumors arise from astrocytes and oligodendrocytes. The majority of glioblastoma are found in the supratentorial brain (frontal, temporal, parietal, and occipital lobes), with rare occurrence in the cerebellum, the brain stem and the spinal cord (3). Glioblastomas may develop in *de novo* patients as a primary glioblastoma or through progression from lower-grade astrocytomas in as secondary glioblastomas (4).

The incidence of glioblastoma is low compared with other cancers (5) such as lung, breast, prostate and colon cancer, but it dwarfs these other tumor types in terms of “average years of life lost” (20.1 years vs. 6.1 years for prostate cancer and 11.8 years for lung cancer) (6), often affecting patients in the prime of their lives. From this perspective, glioblastomas represent a very important problem in oncology, whose grim prognosis has changed little since the 1970s (7).

The surgical approach, if feasible, is the initial mainstay of treatment for patients with newly diagnosed glioblastoma. However, glioblastoma is a highly diffusive, invasive and vascularized tumor, and is not curable with surgical intervention. Therefore, concomitant and adjuvant temozolomide (TMZ), combined with radiation therapy has become established as the standard of care after surgery (8), with high dose steroids (dexamethasone) often prescribed to reduce vasogenic edema. FDA approved treatments for newly diagnosed glioblastomas are limited and include the Optune portable device, also known as Tumor Treating Fields (TTFs), which delivers an electric field to the tumor (9).

Unfortunately, GBM recurrence is inevitable and virtually all patients will relapse, with the majority of relapses occurring centrally within 2 cm of the original gadolinium-enhanced mass on MRI (10). This accounts for its unfavorable prognosis, and, despite decades of investment and research, there is still an unmet medical need for a more efficacious treatment (11). Extracranial metastasis are rare, occurring in less than 2% of patients. This is thought to be due, in part, to the dura mater and the thickened basement membrane of blood vessels that can prevent hematogenous and lymphatic spread, and also because central nervous system (CNS) tumor cells lack extracellular matrix protein required to invade surrounding connective tissue (12). Quality of life in these patients is progressively and significantly impacted due to the emergence of debilitating symptoms arising from infiltrative tumor growth far into functionally intact brain tissue that restricts and disrupts normal day-to-day activities (13).

For all these reasons, the identification and integration of new treatment options that enhance the cytotoxic effects of the standard-of-care radiation/temozolomide regimen constitute an urgent unmet medical need.

## Classification and Histology

The WHO (World Health Organization) Classification of Tumors of the Central Nervous System is the current international standard for the nomenclature used for the classification of gliomas. It designates gliomas according to level of histologic malignancy and mitotic activity (14). Grades I and II gliomas are referred to as low-grade due to their low proliferative potential, while grade III and IV gliomas are high-grade because of their high proliferative rate and aggressive phenotype. As the most aggressive, invasive and undifferentiated type of tumor, glioblastoma is classified as Grade IV.

Glioblastoma were previously classified into 4 subtypes: 1) proneural, 2) neural, 3) classical, and 4) mesenchymal, with the proneural subtype possibly conferring a more favorable prognosis in younger patients. However, recent analysis of transcriptomes of glioma have revealed 3 subtypes strongly enriched with mRNAs associated with classical, proneural and mesenchymal subtypes, but not with the neural subtype, suggesting that this subtype may have arisen from contamination of the original samples with nontumor cells (15).

Over time, molecular biomarkers have played an important role in histological typing and diagnosis of glioblastomas (16), as well as for predicting survival and response to therapy. The best-known biomarker in this disease is the methylation status of the promoter for MGMT (O6-methylguanine-DNA methyltransferase). Epigenetic silencing of MGMT by methylation of the promoter is a favorable prognostic indicator and is associated with increased overall survival (OS) in patients treated with radiation therapy and alkylator chemotherapy (e.g. TMZ) (17, 18). The median OS of patients with MGMT-methylated tumors is 22-26 months compared with non-MGMT-methylated tumors of 12-15 months, respectively (17). Importantly, alteration in MGMT in MGMT-deficient glioblastoma cells has been implicated in acquired TMZ resistance in several ways, including modulation of DIP2A/MGMT signaling by Fstl1 (19), IKBKE-induced upregulation of MGMT (20) and regulation of MGMT expression by miRNAs resulting in degradation of MGMT mRNA before translation (21).

Another clinically relevant biomarker is the mutation status of IDH (isocitrate dehydrogenase)1 and IDH2, with approximately 10% of patients with glioblastoma (14) expressing a mutation in either of these genes, which is an early genetic event in gliomagenesis. Mutant IDH1 is a metabolic marker of secondary glioblastoma because of its ubiquitous expression in lower grade gliomas that eventually progress to glioblastoma. Given the increasing importance of molecular subtyping in glioblastoma (22), the WHO has classified glioblastoma into glioblastoma, IDH-wildtype, glioblastoma, IDH-mutant, and glioblastoma not otherwise specified (NOS) (14). While the WHO classification system has historically served as the primary source of updates on diagnostic classes, grades and criteria, in an effort to more rapidly integrate advances in understanding of brain tumor molecular pathogenesis into practice, cIMPACT-NOW (the Consortium to Inform Molecular and Practical Approaches to CNS Tumor Taxonomy) was established.

Examples of recent cIMPACT-NOW activities include recognition that the lack of an IDH mutation alone is insufficient for designating a glioma as WHO Grade IV. Therefore, the cIMPACT-NOW Update 3 provided diagnostic criteria for “Diffuse astrocytic glioma, IDH-wildtype, with molecular features of glioblastoma WHO Grade IV,” and recommended that histologic grade II and III IDH-wildtype diffuse astrocytic glioma that contain a high level of EGFR amplification, the combination of whole chromosome 7 gain and whole chromosome 10 loss, or TERT promoter mutations, correspond to WHO grade IV and be referred to as diffuse astrocytic glioma, IDH-wildtype, with molecular features of glioblastoma, WHO grade IV (23). Furthermore, they also concluded that subsets of IDH-wildtype diffuse astrocytic glioma with specific molecular signatures have better outcomes and should not be given high grade designation, including those with MYB/MYBL or BRAF mutations (23). cIMPACT-NOW update 1 clarified the use of the term NOS (Not Otherwise Specified) and proposed the use of NEC (Not Elsewhere Classified) (24). cIMPACT-Now Update 6 defined astrocytoma, IDH-mutant as diffusely infiltrative astrocytic glioma with microvasculature proliferation or necrosis with a CDKN2A/B homozygous deletion, connotating grade IV (25).

The clinical significance of IDH in glioblastoma is related to its function in the Krebs Cycle. IDH normally decarboxylates isocitrate to α-ketoglutarate (α-KG) and produces NADPH, which, in turn, reduces GSSG to glutathione (GSH) (22, 26). Mutated IDH instead catalyzes the conversion of α-KG to α-hydroxyglutarate (α-HG), a putative oncometabolite (27), at the expense of NADPH production. While, IDH mutations contribute to tumorigenesis, they also are associated with a better prognosis, possibly as a result of decreased antioxidative levels of NADPH, and by extension, GSH, which render the tumor more radio- and chemosensitive (28). Of note, IDH mutations are virtually absent in the elderly (29).

Besides IDH, secondary glioblastomas are characterized by TP53, PDGF and ATRX mutations, as well as loss of chromosome 19q (30), while in the majority of IDH-wildtype, epidermal growth factor receptor (EGFR) overexpression, phosphate and tensin homolog (PTEN) mutations, and loss of chromosome 10q are common (31).

**Figure 1:**
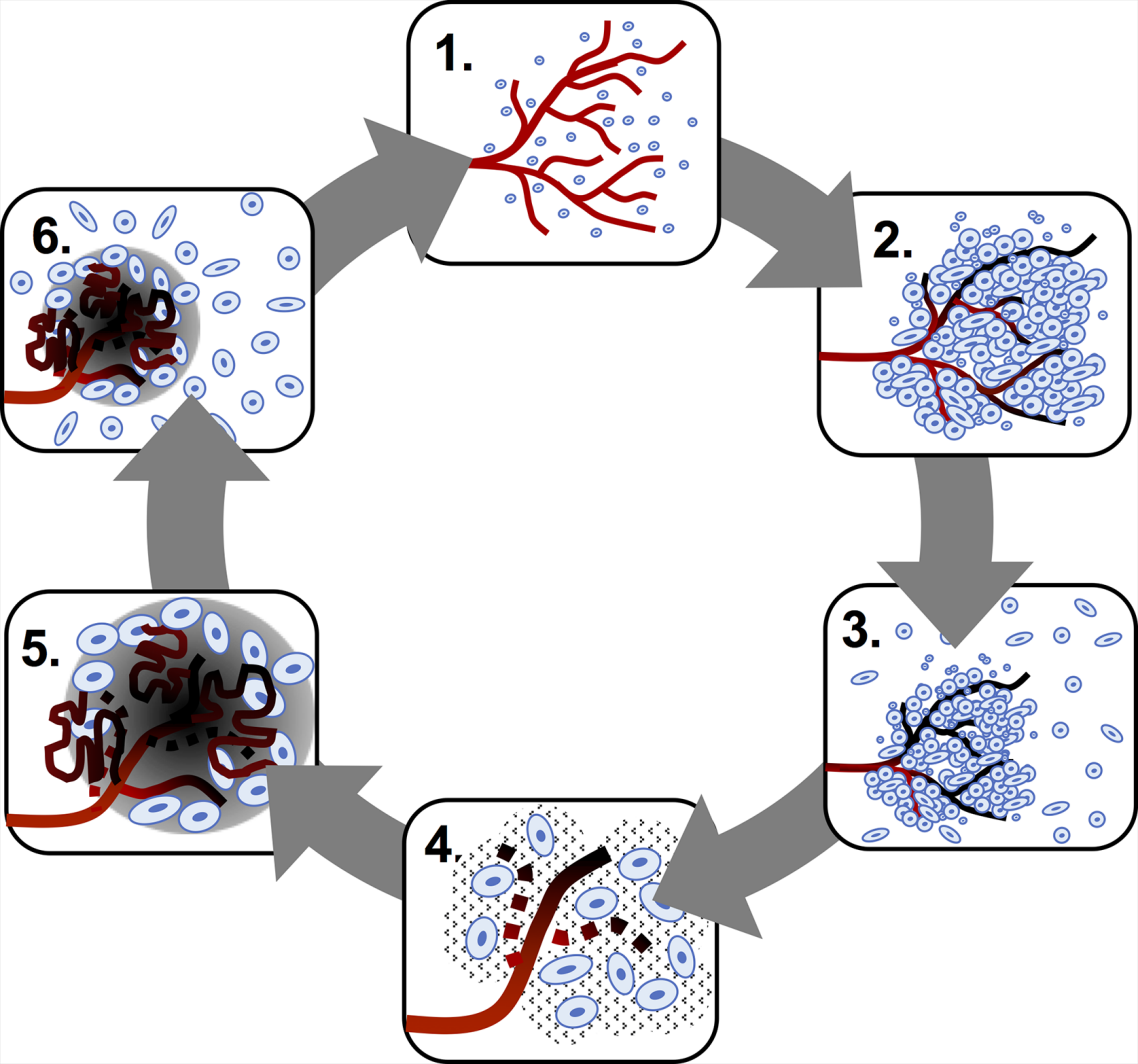
Hypoxia-neovascularization cycle in glioblastoma. The steps below are as follows: 1. Glioma cells consume oxygen provided by the functional vasculature. 2. Endothelial injury, prothrombotic factors and increased mechanical pressure in regions of high glioma cell density induce vaso-occlusion and necrosis. 3. Perivascular glioma cells switch to a “go” phenotype based on presence of hypoxia. 4. Pseudopalisading glioma cells secrete pro-angiogenic factors. 5. Pro-angiogenic factors stimulate the formation of aberrant, highly permeable neovasculature, which results in more hypoxia and accelerated progression. 6. Pseudopalisading cells migrate to a new vasculature where the cycle begins anew.

**Figure 2:**
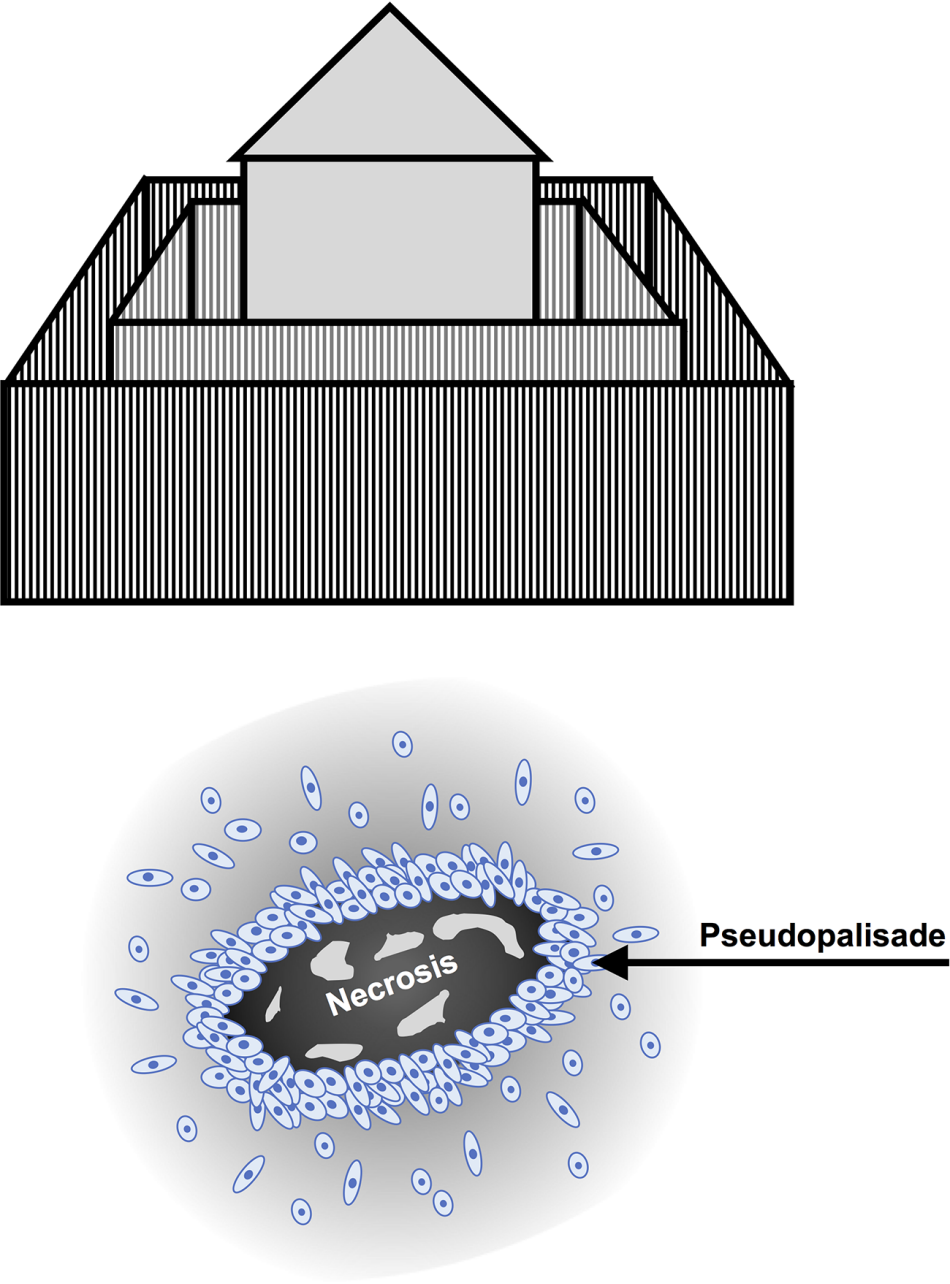
Palisades and pseudopalisading cells. Palisades are defined as a protective layer, similar to a fence or perimeter of wooden stakes or iron railings. (top figure) In glioblastoma, pseudopalisades or “false palisades” are dense migratory zones of cells in picket-fence or perimeter formation, which surround necrotic tissue; hence the term, “pseudopalisading necrosis”. The pervasive hypothesis is that pseudopalisading cells are “microenvironmental migrants” that co-localize in search of better oxygenated conditions due to the presence of vascular collapse and necrosis (40). (bottom figure)

Conventional histologic hallmarks of glioblastoma include cellular polymorphism, nuclear atypia, a high mitotic index (14), pseudopalisading necrosis (32) and microvascular hyperplasia or neovascularization. Glioblastoma is a highly hypoxic and prothrombotic (33) tumor, which promotes compensatory neovascularization driven by the secretion of pro-angiogenic factors, resulting in local vascular hyperplasia, stasis and thromboembolic vascular occlusion that, in turn, augment the hypoxia and accelerate progression. (**Figure 1**) Pseudopalisades, a pathognomic feature of glioblastoma, are densely-packed rows of cells that encircle hemorrhagic necrotic foci in which are found a multitude of thrombosed vessels (34). This local alignment and aggregation of cells is thought to represent a coordinated mass migration event, away from a nutrient- and oxygen-impoverished microenvironment (35). (**Figure 2**)

According to the Go-or-Grow hypothesis, cells can either migrate or proliferate, but not do both, which accounts for the relative quiescence of pseudopalisading cells (36). Those cells that do not migrate undergo apoptosis, eventually leading to a zone of central necrosis. These repeated cycles of angiogenesis, vascular collapse and cellular migration/infiltration contribute to resistance, making treatment of these tumors difficult (37), as tumor cells that have spread far and wide from the main tumor in search of a more hospitable microenvironment around new vasculature make the tumor deeply infiltrative. Interestingly, hypoxia is only marginally present in low grade gliomas, where the vasculature remains largely intact (38), which suggests that low oxygenation created by the tumor itself maybe a driving force responsible for progression and aggressiveness (39).

## Epidemiology and Causative Factors

Glioblastoma, which accounts for about 15% of all CNS cancers (41), is an orphan disease, with less than 20,000 cases in the United States. Nearly half of patients with a new diagnosis of glioblastoma are over age 65, although glioblastoma can occur at any age including childhood (42). Of note, survival markedly decreases with advancing age (41). The tumor is twice as common in whites as in Africans and African-Americans, with a lower incidence in Asians and Native Americans, and slightly more males than females affected (43). No definitive links with smoking, diet, cellular phones, environmental exposures or electromagnetic fields have been identified (44). In 5% of cases a family history is reported and rare syndromes, such as Li-Fraumeni syndrome, neurofibromatosis I and II, Turcot’s syndrome, Ollier disease and Maffucci syndrome have been associated with elevated risk (45, 46). While ionizing radiation is a well-established risk factor, only a minority of presumed radiation-induced cranial tumors are glioblastomas (47). One possible driver of gliomagenesis that has been proposed is infection from CMV or EBV since both viruses have been detected in glioma samples, but it is presently unknown whether this is a causative or epiphenomenal association (48, 49).

## Clinical Presentation and Diagnosis

The clinical presentation of glioblastoma varies depending on the size and location of the tumor as well as the degree of peritumoral edema (50). The most common general symptoms are new-onset headache and seizure, although seizures are more frequent in low grade glioma (51). Focal symptoms such as neurological deficits, and cognitive and personality changes are due to compression and infiltration of normal brain tissue (52). The presence and degree of peritumoral edema correlates with symptomatology. High dose steroids (10-20 mg IV dexamethasone loading dose followed by dexamethasone 4-24 mg in divided doses) (53) are typically administered to reduce tumor-associated vascular permeability and edema.

Gadolinium-enhanced magnetic resonance imaging (MRI) is the gold standard for glioblastoma diagnosis, due to the permeability of immature tumor vessels, which leads to extravascular accumulation of contrast agent with T1 shortening and signal enhancement on T1-weighted images (54). However, true delineation of the tumor is difficult because glioma cells invariably extend beyond the Gadolinium-enhanced abnormality (55).

**Figure 3:**
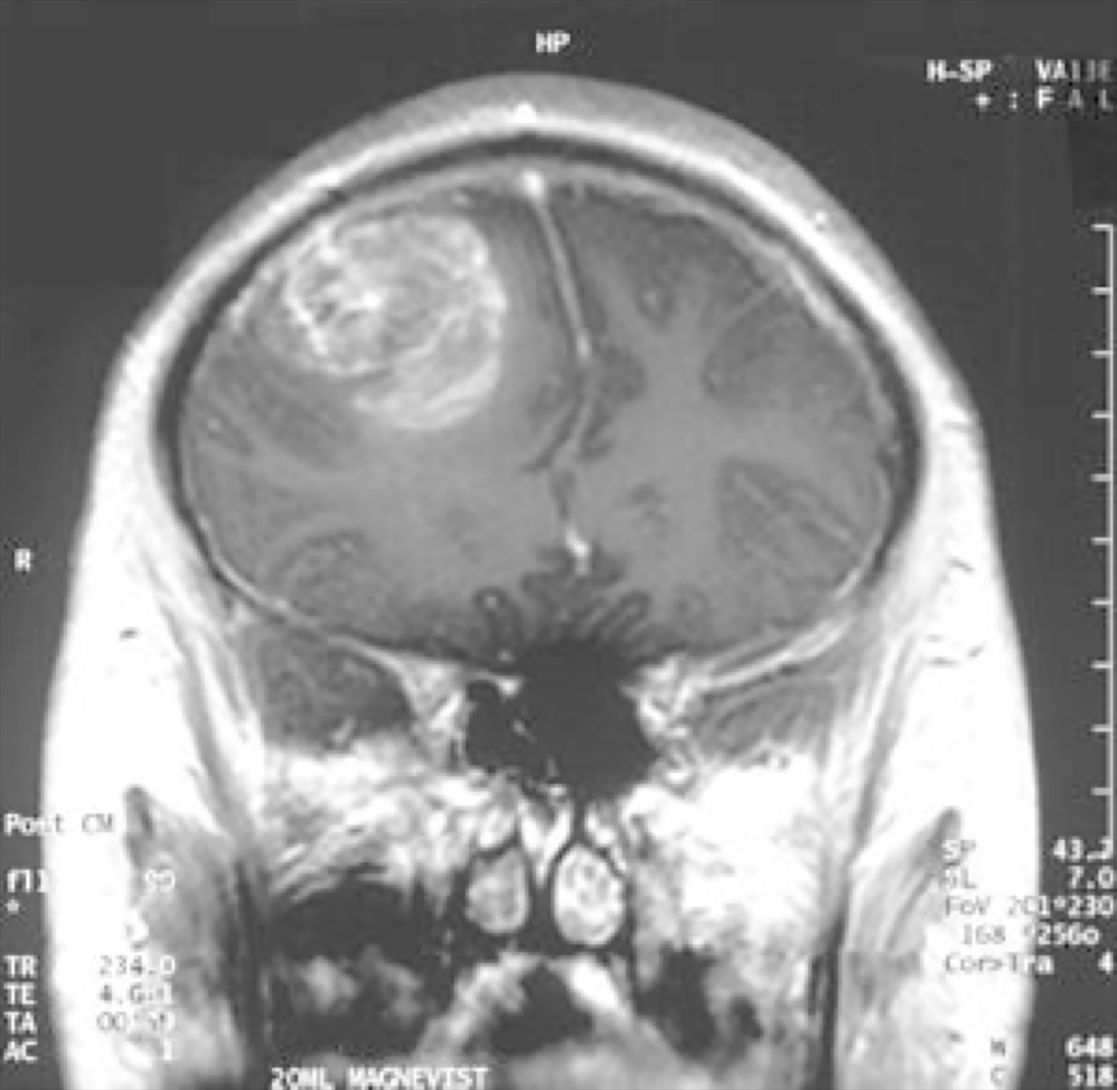
Magnetic resonance imaging (MRI) appearance of glioblastoma: contrast enhancing rind of tumor surrounding a necrotic core (56).

Characteristically, with MRI, glioblastomas appear as a variegated or multiform contrast-enhancing mass(es), with thick, irregular rinds of enhancement surrounding hypointense, necrotic cores. (**Figure 3**) The appearance of necrosis, a hallmark of glioblastoma, is related both to the presence of thrombosed vessels and the rapid proliferative rate of the tumor cells, which leads to an extreme mismatch between accelerated oxygen consumption and the scarcity of blood supply (57). Surrounding vasogenic edema with mass effect, hemorrhage, and distortion or displacement of adjacent structures may also be visible (58).

Radiographic patterns of disease include: local (unifocal disease), distant (second lesion noncontiguous with the primary lesion), multifocal (>2 non-contiguous lesions and/or cerebral spinal fluid dissemination) and diffuse (> 3 cm beyond the primary site) (59).

Advanced neuroimaging techniques, including assessment of perfusion, diffusion and spectroscopy, are very useful for purposes of characterizing glioblastomas at the time of diagnosis and for monitoring response to therapy and recurrence post treatment (60–64). Magnetic resonance spectroscopy (MRS) can be used to study metabolic changes in brain tumors. For example, use of proton MRS for biochemical profiling and measurement of phosphocholine, creatine and N-acetylaspartate, as well as associated relevant concentration ratios, have a role in the diagnosis of glioblastoma (63). Clinical applications include use of 1H-MRS for the differentiation of glioma recurrence from radiation necrosis (65). This approach is not only applicable to Hydrogen, but also to many other nuclei or isotopes (66). In diffusion-weighted imaging, water molecule diffusion is characterized by an apparent diffusion coefficient (ADC) (64). In glioblastoma, ADC is useful for early characterization of tumor, as there is an inverse correlation between ADC and grade (67), and may also help to detect early recurrence, especially in non-enhancing regions of the brain (68). Another imaging technology, diffusion tensor imaging, generates a mathematical model of diffusion in 3-D (66), and can be useful for the detection of tumor infiltration of adjacent normal appearing white matter (69). Perfusion weighted imaging allows for imaging of neovascularization, with assessment of blood flow, volume and leakage (70), and has been useful for prognostication in glioblastoma (71), assessment of treatment response, and differentiation of recurrence/progression from stable disease (72). Lastly, a new imaging approach, chemical exchange saturation transfer (64) allows for detection of specific molecules present at very low concentrations.

## Therapeutic Management

### Treatment

Treatment of glioblastoma is quadripartite: 1) surgery 2) adjuvant radiotherapy and TMZ 3) maintenance TMZ and 4) alternating electric fields

### Surgery

The bedrock of glioblastoma treatment is gross total resection or subtotal resection to immediately relieve the tumor-associated mass effect, delay recurrence, and obtain tissue for molecular testing (73). Complete resection is currently impossible due to the diffuse infiltrative nature of the disease and the potential for iatrogenic damage to intact brain tissue as a result, especially when the tumor is located in or near the eloquent cortex, critical areas whose removal permanently compromises sensory processing, linguistic ability, and/or motor function (74). The presence of residual disease is responsible for the inevitable subsequent disease progression or recurrence, usually six months after surgery (75).

Nevertheless, aggressive resection is warranted on the basis of several studies, which have demonstrated that the smaller the residual tumor burden, the longer the progression-free survival (PFS) and OS, which makes the extent of resection a crucial prognostic factor (76–78). However, despite the introduction of several new surgical techniques and innovations (79), including neuronavigation, fluorescence, and intraoperative imaging (MRI, CT, ultrasound) to maximize visualization of tumor borders and optimize the extent of resection, while minimizing damage to adjacent normal brain, the prognosis and the quality of life for glioblastoma patients remains poor (80).

### Chemoradiation Therapy

After maximal surgical resection, the standard of care is post-operative radiotherapy (81) to a field encompassing the primary tumor with a 2-3 cm margin. Radiation is commonly given over six to weeks with three-dimensional conformal beam or intensity-modulated radiotherapy (RT) (2 Gy per daily fraction (Monday to Friday) for a total dose of 60 Gy) with concomitant TMZ chemotherapy at a dose of 75 mg per square meter of body-surface area per day, 7 days per week followed by maintenance TMZ at a dose of 150-200 mg per square meter for 5 days during each 28-day cycle for up to 6 adjuvant chemotherapy cycles. Because the addition of TMZ to surgery and radiotherapy for newly diagnosed glioblastoma resulted in a clinically meaningful and statistically significant survival benefit with minimal additional toxicity, concomitant TMZ and radiotherapy followed by 6 cycles of adjuvant TMZ was adopted as the new standard of care for newly diagnosed glioblastoma (8). This practice change was based on a Phase 3 EORTC 26981-22981/NCIC CTG trial in patients with WHO performance status of 2 or more. In this study median PFS was 6.9 months in the TMZ arm vs. 5.0 months with radiation therapy arm, and a median survival of 14.6 months vs. 12.1 months, respectively. Of note, in the United States, non-progressive patients have been treated with TMZ for up to 12 cycles or more (82). Importantly, a recent phase II trial of continuing TMZ beyond 6 adjuvant cycles was associated with more toxicity, without any additional benefit in 6 month PFS (83).

The Stupp protocol (8), based on the EORTC-NCIC trial paper discussed above, established the primacy of TMZ and RT to RT alone. The analysis of this trial also correlated outcomes with MGMT (O6-methylguanine-DNA methyltransferase) methylation status. As an alkylating agent, temozolomide methylates DNA at the O6, N3, and N7 positions, which, if unrepaired, leads to DNA strand breaks (84). MGMT is a DNA repair enzyme located on chromosome 10q26 whose methylation is associated with loss of MGMT expression (85) and, hence, enhanced sensitivity to TMZ as a result of reduced DNA-repair activity (86). Conversely, an unmethylated MGMT enzyme results in high activity of MGMT and reduced activity of TMZ. Therefore, methylated MGMT is a favorable prognostic factor (87). More recently, in an effort to improve upon the standard of care regimen using TMZ, a phase 3 trial was performed comparing the combination of lomustine and TMZ vs. standard TMZ in patients with newly diagnosed glioblastoma with methylated MGMT promoter, with a median survival of 31.4 m vs. 48.1m (88).

In elderly or poor performance patients, hypofractionated radiotherapy (e.g., 40 Gy/15 Fractions) has emerged as a viable alternative to standard radiation therapy (e.g., 60 Gy/30 Fractions) on the basis of several studies, which collectively indicate that while both confer a similar survival benefit, hypofractionated radiation therapy is associated with less neurotoxicity and steroid administration (89).

Given the potential role of hypoxia in the biology of glioblastoma, there has been considerable interest in anti-angiogenesis therapies, but concern that long term treatment with agents such as bevacizumab might increase tumor hypoxia and result in a more infiltrative phenotype (90). Two Phase 3 trials of bevacizumab in the first line setting (91, 92), demonstrated improvement of PFS without a corresponding improvement in OS and, on this basis, bevacizumab is not administered in the first line therapy. Moreover, bevacizumab was associated with pseudoresponse, a term which refers to a rapid regression of enhancement due to vascular regression with a corresponding increase of invasiveness due to induction of hypoxia (93).

A number of new combination regimens incorporating immunotherapy have been conducted in the newly diagnosed setting. Immunotherapy is a potentially promising strategy due to the presence of a functional CNS lymphatic system and an altered blood brain barrier, which facilitate immune cell trafficking in and out of the CNS (94). Of note, glioblastoma is an “immune cold tumor”, meaning that it is not very immunogenic, with a very low mutational burden, and a highly immunosuppressive microenvironment, characterized by a low level of T cell infiltration (95). Nevertheless, several studies have shown that neoadjuvant PD1 blockade can modulate the glioblastoma microenvironment resulting in enhanced anti-tumor immune responses, elevated expression of chemokine transcripts, increased immune cell infiltration into tumors, and greater T cell receptor clonal diversity (96–98). The question remains whether or not these biological effects will translate into clinical benefit. Recent and ongoing trials combining immunotherapy with standard of care therapy are addressing this question. Unfortunately, Phase 3 clinical trials with nivolumab in newly diagnosed glioblastoma patients with unmethylated MGMT promoter status (CheckMate-493; NCT02617589) combined with radiation therapy (99), and in patients with methylated MGMT promoter status (CheckMate-548; NCT02667587) (100) combined with standard of care radiation therapy and TMZ (vs. standard of care treatment) failed to demonstrate a significant improvement in OS in CheckMate-498, and failed to meet the PFS primary endpoint in CheckMate-548. However, CheckMate-548 will be continued to allow the other primary endpoint, OS, to mature. Another immunotherapy combination trial, PERGOLA (NCT03899857) is evaluating the efficacy and toxicity of adding pembrolizumab to concurrent radiation therapy and TMZ in newly diagnosed glioblastoma patients. One caveat with immunotherapy in this setting is that the frequent use of high-dose corticosteroids to attenuate cerebral edema may serve to dampen anti-tumor immune responses.

### Tumor Treating Fields

In October 2015, Optune^®^, a noninvasive portable device, which delivers alternating electric fields (TTFs) to disrupt cell division (101), was approved by the U.S. Food and Drug Administration (FDA) as an adjunct to TMZ in first-line glioblastoma on the basis of a Phase 3 trial called EF-14 in which the median OS and PFS at interim analysis (n=315) was 19.6 months for TTFs/TMZ vs. 16.6 months for TMZ alone (HR 0.75; log-rank p = 0.034) and 7.1 months for TTFs/TMZ vs. 4.0 months for TMZ alone(HR 0.6; log-rank p = 0.0014), respectively (9). In addition, an increase in 5-year survival from 5% to 13% was observed (102). Moreover, TTFs was safe with skin reactions from the application of electrodes to the head as the main adverse event (103). Nevertheless, use has been limited due to cost, patient stigmatization/inconvenience and the novelty of the mechanism (104).

### Recent and Anticipated Results From Clinical Trials

Among some of the most eagerly anticipated results are those from a Phase 3 trial with DCVax-L, an autologous dendritic cell vaccine, with radiation therapy and TMZ. While final results are pending, an interim analysis demonstrated a mean OS of 23.1 months, with 2 and 3 year survival of 46.2% and 25.4%, respectively, with a higher OS in patients with methylated MGMT of 34.7 months vs. 19.8 months. To date DCVax has been safe and well tolerated with only 2.1% SAEs (105). Other promising vaccine approaches include SurVaxM, a peptide vaccine to survivin, with a single arm Phase II trial showing benefit in terms of PFS and OS (106). A prospective randomized trial is planned and expected to open to accrual in 2020.

There was also considerable interest in a Phase 3 trial using the antibody-drug conjugate depatuxizumab mafodotin (ABT-414), which binds activated EGFR, in combination with standard-of-care treatment in patients with newly diagnosed, EGFR-amplified glioblastoma. Unfortunately, an interim analysis failed to show an OS benefit, and the study was stopped for futility (107). EGFR has been thought of as an attractive target, due to EGFR amplification and overexpression in approximately 50% of glioblastoma tumors (108), even though, to date, trials with EGFR-pathway inhibitors have been disappointing (109), likely due to poor BBB permeability with subtherapeutic drug levels. Unfortunately, so far there is no evidence to date of an OS benefit with the addition of anti-EGFR treatments in first line or recurrent glioblastoma (110).

A large number of targeted agents are currently under investigation in early clinical trials (111). An example is the use of poly-(ADP-Ribose)-DNA Polymerase (PARP) inhibitors to enhance the efficacy of standard of care chemoradiation. There is considerable interest, in particular, in the use of veliparib. However, preliminary results with veliparib in the VERTU trial, a randomized Phase II trial in MGMT-unmethylated glioblastoma patients found that the combination of veliparib with chemoradiation was well tolerated, but did not improve outcomes (112). This combination is now being evaluated in a Phase III trial in MGMT-methylated newly diagnosed glioblastoma patients with adjuvant temozolomide, with results expected soon. Another active area of investigation is the use of targeted agents in place of TMZ, While trials replacing TMZ with targeted agents in unselected patients without MGMT promoter hypermethylation have also shown no OS benefit, an important new Phase 1/11a NCT Neuro Master Match umbrella trial is ongoing in newly diagnosed glioblastoma patients with IDH-wild type, without MGMT promoter hypermethylation, with a variety of promising agents in combination with standard radiation therapy (113). In addition, there are a number of promising new therapies and new agents in the pipeline (114, 115) in early preclinical/clinical development, such as TORC ½ inhibitors (116), tumor targeting peptides (117), immunotherapies (118, 119); combination therapies with anti-CD47 (120), and approaches targeting cancer cell metabolism (121).

## Conclusion

Despite decades of research, glioblastoma remains one of the deadliest and most feared of all cancers. Treatment for it has reached a relative impasse with the use of surgery, radiation, TMZ, and TTFs, since the addition of other targeted agents such as angiogenesis and EGFR inhibitors have failed to date to improve survival, and there is no standard treatment for recurrence, which is inevitable. New therapies are desperately needed for first line treatment, with a number of promising therapeutic agent currently in development.

## Author Contributions

BO and SK contributed to the study concept, design, and are guarantor of integrity of the entire study. BO, TR, AO, NS, and SK all contributed to literature search, manuscript preparation, and manuscript editing. All authors contributed to the article and approved the submitted version.

## Conflict of Interest

Author BO is employed by the company EpicentRx. AO is currently employed by InterWest Partners.

The remaining authors declare that the research was conducted in the absence of any commercial or financial relationships that could be construed as a potential conflict of interest.
